# Multidisciplinary Management of Cervical Horizontal Root Fracture: A Case Report and Literature Review

**DOI:** 10.7759/cureus.96808

**Published:** 2025-11-13

**Authors:** Arunoday Kumar, Shamurailatpam Priyadarshini, Thingujam Debica, T. P Devi, Khumanthem Savana, Kshetrimayum Martina

**Affiliations:** 1 Department of Prosthodontics and Crown and Bridge, Regional Institute of Medical Sciences, Imphal, Imphal, IND; 2 Department of Conservative Dentistry and Endodontics, Regional Institute of Medical Sciences, Imphal, Imphal, IND; 3 Department of Orthodontics and Dentofacial Orthopedics, Regional Institute of Medical Sciences, Imphal, Imphal, IND

**Keywords:** cervical horizontal root fracture, fibre post, occlusion, root canal treatment, splint

## Abstract

Cervical horizontal root fractures (CHRFs), although less common than mid-root fractures, present a significant clinical challenge due to their location near the alveolar crest, compromising tooth stability and complicating treatment. This paper reviews the etiology, classification, diagnosis, and current evidence-based management strategies for horizontal root fractures, and a case report on the management of CHRF is presented. Emphasis is placed on decision-making between conservative, prosthetic, orthodontic, or surgical treatment, and prognosis factors that influence long-term outcomes. Management of CHRF requires accurate diagnosis and a multidisciplinary strategy tailored to the specific fracture type and patient needs. Collaboration among dental specialties enhances prognosis and patient outcomes.

Though CHRF cases are considered to have a poor prognosis, a carefully, well-planned interdisciplinary treatment procedure and various therapeutic techniques and approaches can be employed to promote healing and preserve the teeth with CHRF without undergoing extraction.

This case report presents the successful management of a tooth with CHRF through a well-planned endodontic and prosthetic approach. The two fractured fragments were approximated, stabilized, and supported using a resin-bonded fiber post. Owing to its modulus of elasticity similar to that of dentin, along with superior flexural, tensile, and fatigue strength, the fiber post offers enhanced resistance to root fracture. Following root canal therapy and tooth preparation, the tooth was restored and splinted with an all-ceramic bridge involving the adjacent teeth. In a diabetic patient with well-controlled blood glucose on medication, healing is anticipated to be fair. Moreover, post-cementation, the restored tooth was kept out of occlusion during functional movements to minimize mechanical stress on the tooth and restoration, thereby promoting healing and optimal biomechanical stability.

## Introduction

Root fractures are uncommon dental injuries, constituting approximately 0.5-7% of all dental traumas [1]. Root fractures are categorized based on their location along the root as apical, middle, or cervical third fractures, corresponding to the apical, middle, and cervical portions of the root, respectively. Mostly, horizontal root fractures occur in the middle third of the root, but fractures occurring in the cervical third, though less frequent, often result from direct trauma and may involve displacement, pulp necrosis, or periodontal compromise [2,3]. Effective management is crucial for preserving the natural dentition and maintaining aesthetics and function [4].

The prognosis of horizontal root fracture depends on various factors, including the location and extent of the fracture, displacement of the coronal fragment, stage of root development, and the time elapsed between injury and treatment [5]. Accurate diagnosis is essential and often requires careful radiographic evaluation and interpretation [6]. Radiographic imaging techniques employed for detecting root fractures would be cone beam computed tomography (CBCT) or intraoral periapical radiograph (IOPAR). CBCT imaging demonstrates higher sensitivity in detecting both vertical and horizontal root fractures, regardless of the involvement of adjacent structures. Its specialized design allows for the accurate and undistorted visualization of dental anatomy [6]. However, in IOPAR, two radiographs taken at different vertical angulations enhance the detection of horizontal root fractures and are therefore also recommended for accurate diagnosis [6].

Cervical horizontal root fractures (CHRFs) hold significant clinical importance due to their proximity to the gingival margin and alveolar crest. This location often compromises the stability of the coronal fragment, increasing the risk of pulpal necrosis and periodontal involvement from bacterial contamination. Furthermore, restorative management is challenging because of the limited remaining root structure (ferrule) available for retention. Such fractures, commonly occurring in the anterior region, also affect aesthetics and function. Consequently, CHRFs generally carry a poorer prognosis compared to middle or apical third fractures and may require complex, multidisciplinary management for optimal outcomes.

Treatment options range from conservative approaches such as repositioning and splinting to more complex procedures, including endodontic therapy, orthodontic extrusion, or even extraction and prosthetic rehabilitation [7,8].

Previous case studies have reported the use of glass fiber posts possessing a dentin-like modulus of elasticity and high flexural strength, along with resin cements that offer improved bonding ability. This combination has enabled clinicians to conservatively approximate, stabilize, and support fractured teeth [8].

Evidence from the available literature further highlights that complex traumatic injuries, such as CHRF, can be successfully managed and frequently demand a multidisciplinary approach involving endodontists, prosthodontists, periodontics, and, in some cases, orthodontists and oral surgeons [7]. Collaborative treatment planning ensures that both functional and aesthetic outcomes are addressed, particularly in the anterior region where patient expectations are high.

This paper presents a case report of a controlled diabetic patient with CHRF in a maxillary canine, successfully managed through an interdisciplinary approach, resulting in a favorable prognosis. The clinical decision-making process, treatment steps, and follow-up outcomes are discussed in the context of current literature to underscore the importance of integrated care in such complex dental trauma cases.

## Case presentation

**Figure 1:**
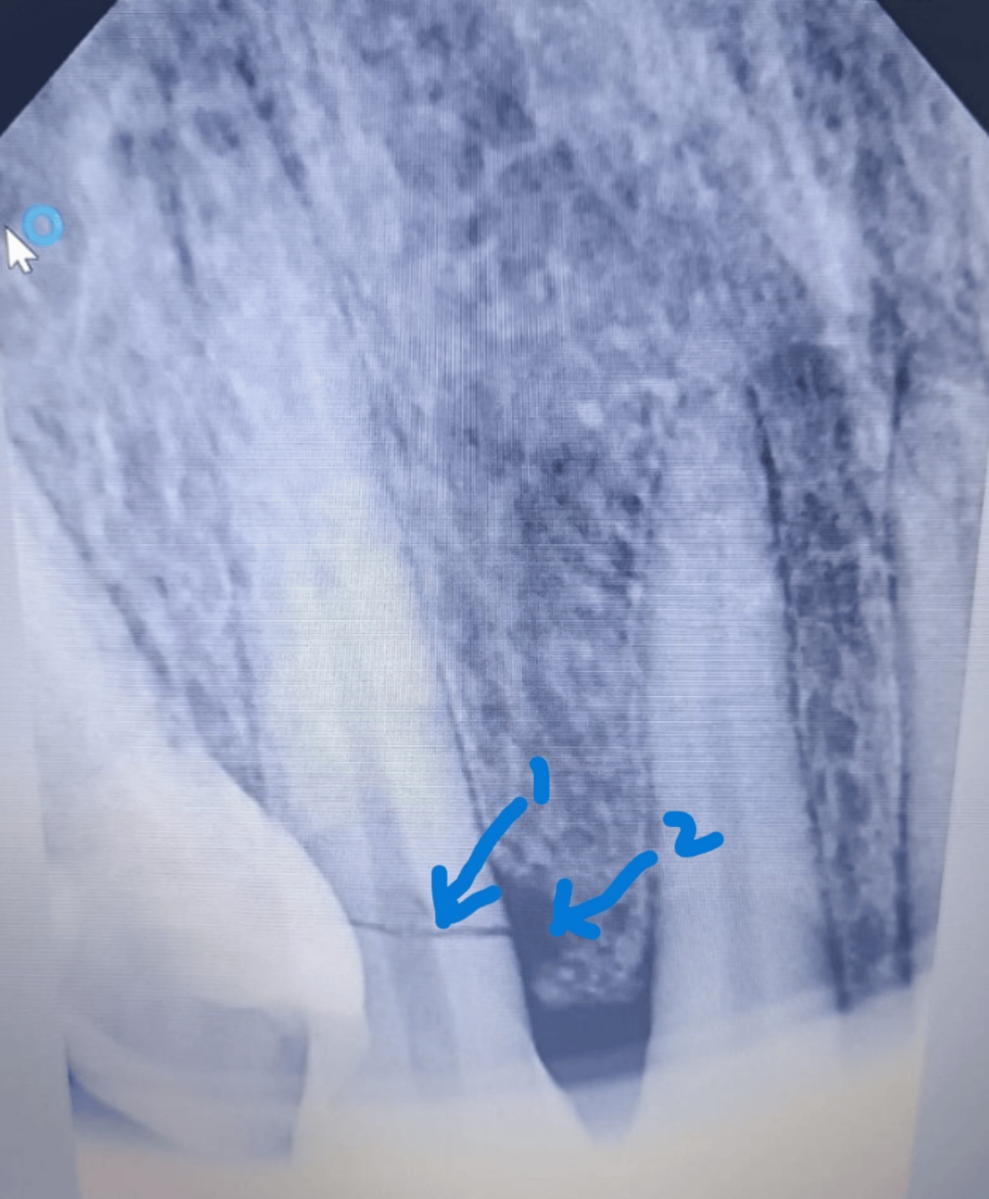
Pre-operative IOPAR showing CHRF (highlighted with arrow 1) with crestal bone loss (highlighted with arrow 2) with respect to tooth #13. IOPAR: intraoral periapical radiograph; CHRF: cervical horizontal root fracture

A female patient in her 40s reported to the Department of Prosthodontics and Crown and Bridge, Regional Institute of Medical Sciences (RIMS), Imphal, India, complaining of pain and slight mobility in the upper right front region for the past two weeks, following traumatic injury. The patient is a controlled diabetic and is currently on medication. She has a history of chewing areca nut and tobacco for the past six years, though she has recently reduced her consumption. On clinical examination, tooth #13 was Grade I mobile and was tender on percussion. A fracture of the palatal cusp was observed with respect to tooth #15, and generalized extrinsic stains were present. On radiographic examination, an IOPAR of tooth #13 revealed CHRF with alveolar crestal bone loss and widening of the periodontal ligament (PDL) space in the apical region (Figure 1).

A cold test was performed with ice sticks to evaluate the vitality status of tooth #13. The tooth did not show any response or sensitivity suggestive of irreversible pulp damage necessitating root canal treatment (RCT). Although the pulp was necrotic, the tooth exhibited tenderness on percussion, indicating the presence of apical periodontitis. After examining the case both clinically and radiographically, a final diagnosis of CHRF with apical periodontitis was made with respect to tooth #13.

Treatment plan

The treatment plan included RCT followed by post and core rehabilitation using a resin fiber post for tooth #13. The patient was referred to the Department of Conservative Dentistry and Endodontics for the RCT procedure.

Endodontic management

**Figure 2:**
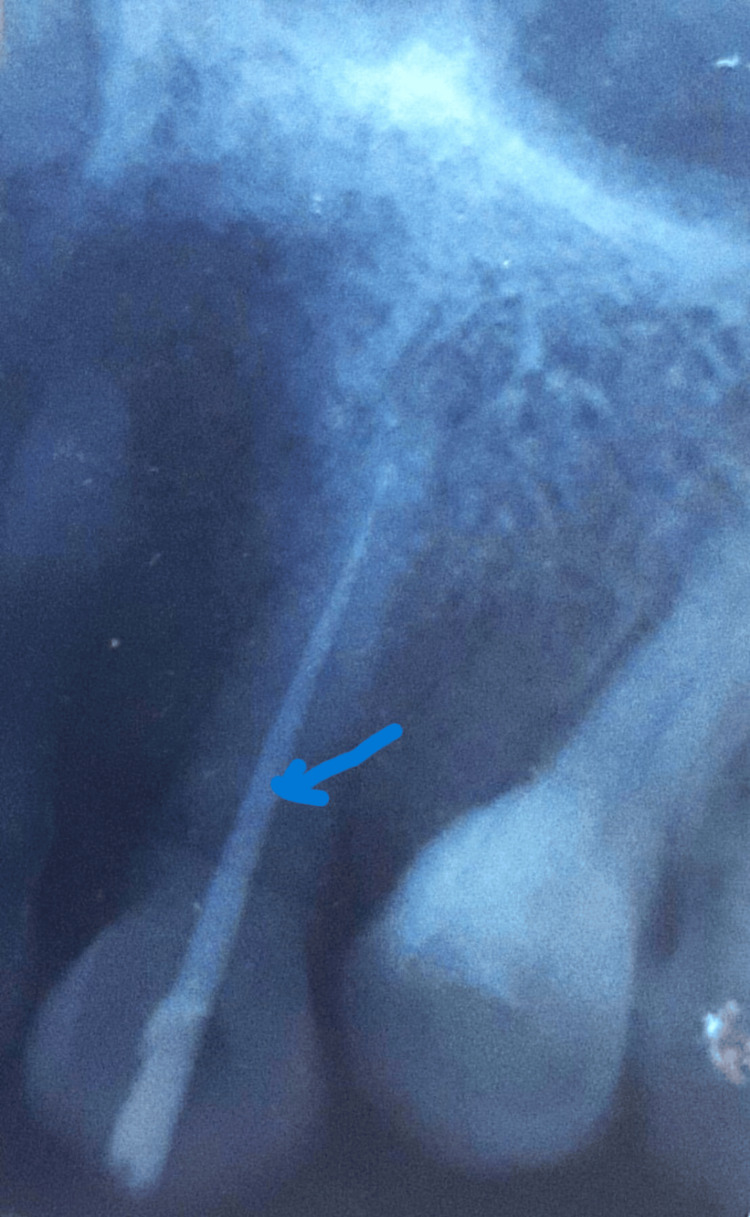
Post-operative IOPAR showing RCT tooth #13 (post obturation) as highlighted with arrow. RCT: root canal treatment; IOPAR: intraoral periapical radiograph

In this case, rubber dam isolation was performed to maintain an aseptic environment, followed by chemomechanical preparation of both the coronal and apical fragments. The canals were then dressed with calcium hydroxide and subsequently obturated. The step-back technique was employed for canal shaping, accompanied by passive irrigation using 3% sodium hypochlorite, normal saline, 17% ethylenediaminetetraacetic acid (EDTA), and a final rinse with 2% chlorhexidine. Calcium hydroxide was placed as an intracanal medicament, and the access cavity was temporarily sealed with Cavit (3M ESPE, Seefeld, Germany). After two weeks, at the recall appointment, obturation was performed using gutta-percha and AH Plus sealer (Dentsply DeTrey GmbH, Konstanz, Germany) with the lateral condensation technique (Figure 2).

The lateral condensation technique was selected due to its simplicity, conservative canal preparation, and precise placement of gutta-percha and sealer within the post space, minimizing the risk of void formation. Calcium hydroxide was chosen for its well-documented antimicrobial properties and its ability to promote remineralization.

Prosthodontic management

**Figure 3:**
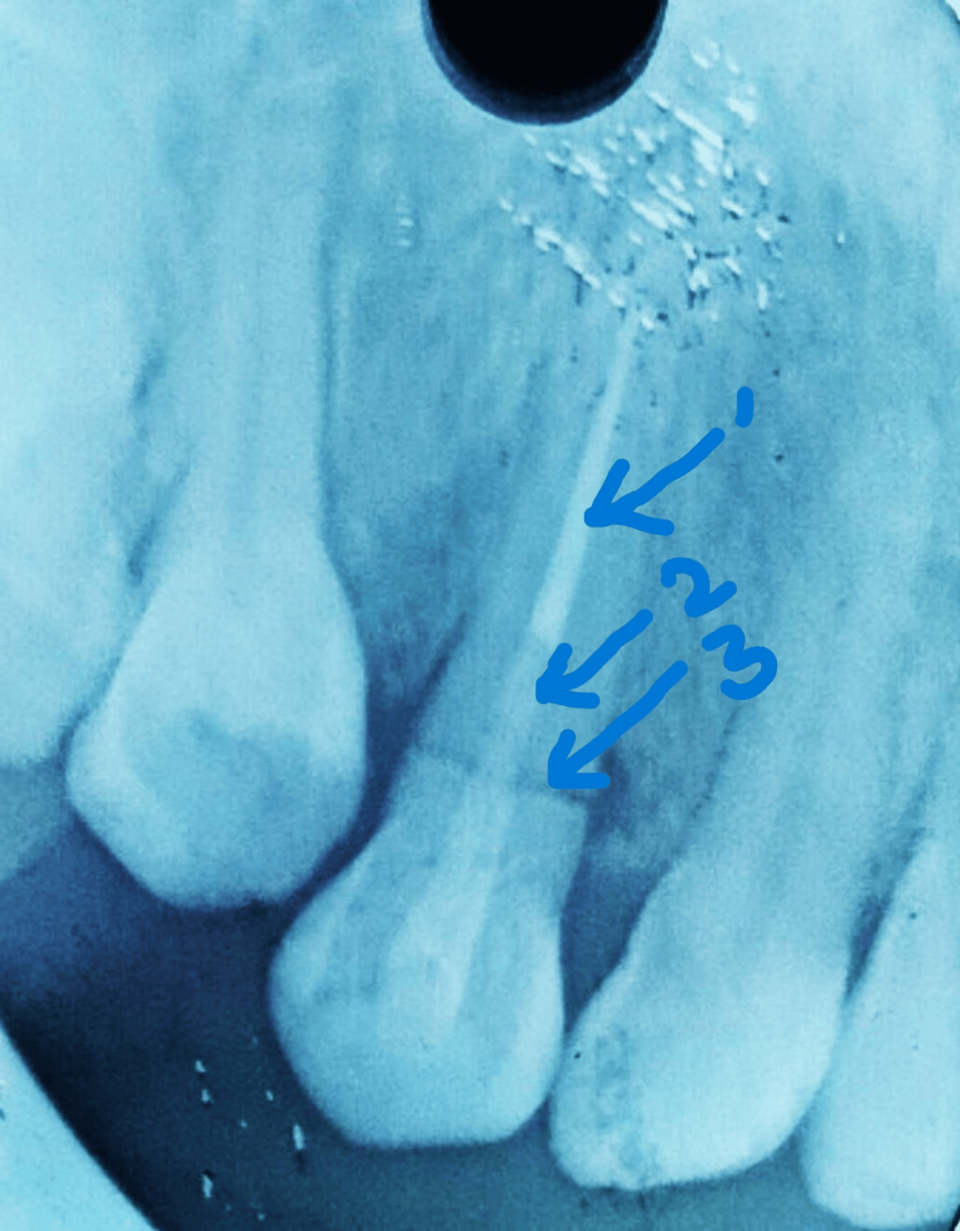
Postoperative IOPAR showing a fiber post incorporated into tooth #13 beyond the fracture line (indicated by arrow 2), stabilizing the fractured crown and root fragments (indicated by arrow 3), with an intact apical seal provided by the obturation material, gutta-percha (indicated by arrow 1). IOPAR: intraoral periapical radiograph

One week after completion of the RCT of tooth #13, post and core build-up were done using a fiber post in order to reapproximate and provide stability and support to the fractured crown and root fragments (Figure 3).

**Figure 4:**
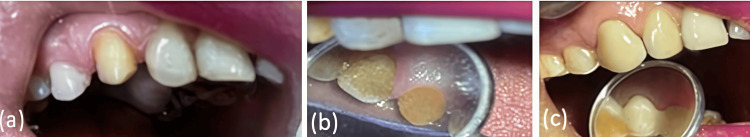
Tooth preparation and final prosthesis: (a) Tooth #13 prepared for a PJC; (b) tooth #14 prepared for a three-fourth crown; (c) tooth #15 prepared for an inlay restoration, followed by permanent cementation of the bridge. PJC: porcelain jacket crown

Post space preparation for tooth #13 was performed using a Peeso reamer No. 2 (Mani Inc., Tochigi, Japan), extending 8 mm apical to the fracture line. A 15 mm glass fiber post (Angelus, Paraná, Brazil) was then placed, with 8 mm engaging the prepared post space and the remaining 7 mm extending coronally beyond the fracture line into the tooth structure. It was then luted with dual-cure resin cement (ParaCore, Coltene, Altstätten, Switzerland) so as to stabilize both the broken fragments of crown and root. After 10 days of recall, tooth preparation was carried out for a porcelain jacket crown on #13, a three-fourth crown on #14, and an inlay restoration on #15 to restore the fractured palatal cusp (Figures 4a-4c).

Cementation and occlusal adjustment

**Figure 5:**
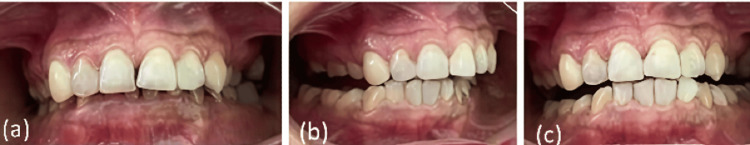
All-ceramic bridge on teeth #13, #14, and #15 cemented with permanent resin cement, shown out of occlusion: (a) centric occlusion; (b) lateral excursive movement; (c) protrusive movement.

The final prosthesis was cemented using permanent resin cement (RelyX™ U200, 3M ESPE, Seefeld, Germany). Occlusion was carefully evaluated and adjusted in centric occlusion, lateral excursive movements, and protrusive movements so as to make canine out of occlusion under various excursive movements of the mandible (Figures 5a-5c).

The prosthesis was intentionally made out of contact with opposing teeth so as to reduce mechanical stress or load on the tooth and restoration, allowing for better biomechanical stability.

Follow-up

**Figure 6:**
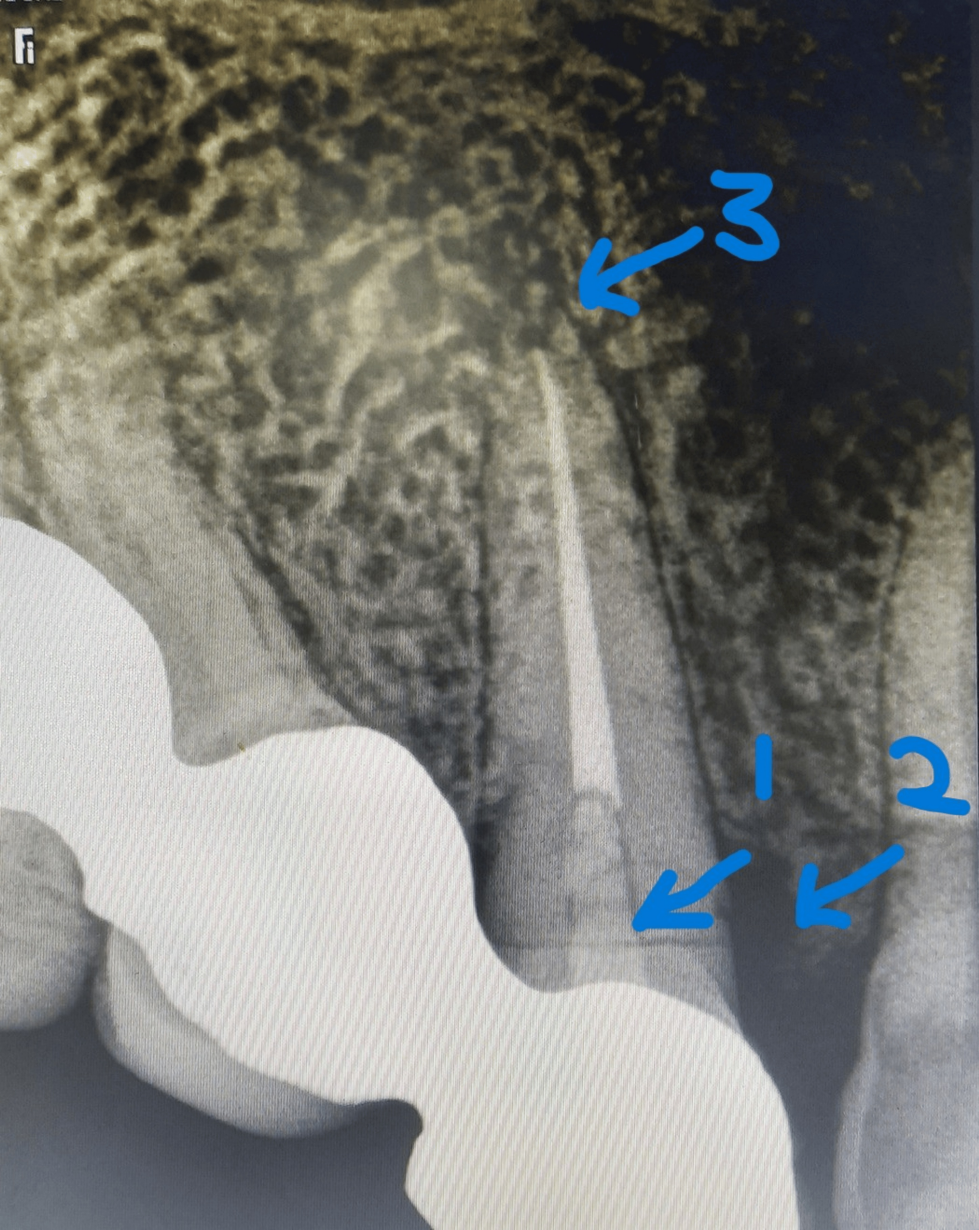
Follow-up IOPAR at six months showing evidence of ongoing healing at the fracture site (indicated by arrow 1) and crestal bone loss at the same site (indicated by arrow 2), with no evidence of periapical radiolucency (indicated by arrow 3). IOPAR: intraoral periapical radiograph

A follow-up of the patient was done at one, three, and six months of interval. At the six-month follow-up, an IOPAR revealed loss of bone in the vicinity of the fracture site, without any evidence of periapical radiolucency (Figure 6). As evident from the radiograph, preoperatively, the fracture line was located 2 mm apical to the alveolar crest, with signs of localized resorption near the fracture site. At the six-month follow-up, the fracture line was observed at the level of the alveolar crest, indicating approximately 2 mm of alveolar bone resorption.

Clinically, there were no signs of mobility, pain, or tenderness. The prosthesis was functioning satisfactorily, and the overall prognosis remained fairly favorable. Informed consent was taken from the patient for the scientific paper publication.

## Discussion

Root fractures can be classified based on location, that is, apical, middle, or cervical third [1,3,5,6]. Based on displacement, they could be with or without coronal segment displacement, and based on communication, there may be either a connection or no connection with the oral environment [1,3,5,6]. Cervical fractures are further subdivided into supra-crestal, which is seen above the alveolar bone, and sub-crestal, seen within or below the bone level [3,5-7].

The aetiology for root fractures can be direct trauma because of sports injuries or accidents [6,7]. They could also be because of pathologic weakening caused due to root resorption or endodontic over-instrumentation [7,8]. Iatrogenic reasons can also lead to root fracture during post-space preparation [8].

On clinical examination, mobility of the coronal fragment, pain or discomfort on percussion, gingival bleeding or pocket formation near the fracture site, and swelling can be seen [7-13]. On radiographic examination, IOPARs or CBCT will reveal the location of the horizontal root fracture [14].

The management of CHRFs depends on the location and extent of the fracture, pulp vitality, mobility, and displacement of the coronal segment, patient’s age, and aesthetic demand.

Management of horizontal root fracture requires an interdisciplinary approach wherein a conservative and endodontic treatment, periodontal, orthodontic, prosthetic management, or surgical intervention may be required [7,8]. Conservative management is required when the fracture is supra-crestal and there is no mobility or displacement of the affected teeth, and the pulp is vital. Semi-rigid splints for four to six weeks are used for stabilization and periodontal healing. The patient should be monitored with regular follow-ups, including pulp vitality testing and radiographic evaluation at three-, six-, and 12-month intervals. Endodontic management is indicated when pulp necrosis occurs, and stabilization of the broken root fragment is adequate. However, in this case, the tooth with CHRF was treated endodontically because the tooth was non-vital as confirmed by the tooth vitality test, and the contamination of the pulp had taken place because of the closeness of the fracture line to the oral environment, and hence tissue repair is also hampered [15-17]. However, RCT for horizontal root fracture in the middle third is not performed, as evidenced in existing literature, as the pulp remains vital in most cases and there is successful healing without undergoing endodontic therapy [5,10,11].

Prosthetic management is done with a post and core to stabilize the broken fragment, followed by crown fabrication and splinting it with the adjacent tooth with a three-fourth crown [4]. A splinted prosthesis was fabricated, considering the micromobility of the maxillary canines and premolars. Since these teeth have similar micromobility in their respective PDL spaces and hence respond similarly to functional loads, no torsional forces were generated, minimizing the risk of prosthesis failure [17].

Treatment modality varies depending on the clinical case presented. If the apical fragment is intact and asymptomatic, then only the treatment of the coronal fragment is required, followed by obturation of the coronal segment with gutta-percha or bioceramic materials [9]. Calcium hydroxide apexification or mineral trioxide aggregate (MTA) plug is done if the apical closure is incomplete [18]. Surgical extraction of the tooth is required when significant coronal mobility or sufficient loss of periodontal structure is seen, thereby leading to grade III mobility or if persistent symptoms or infection are encountered [6]. Extraction of the coronal fragment is indicated only if the coronal part exhibits Grade III mobility, with retention of the apical root (decoronation), followed by root extrusion (orthodontic or surgical) to bring the fracture line to a supracrestal level, so as to prosthetically rehabilitate it with a post and core and crown [7]. Apicoectomy is indicated if apical pathology exists. Extraction of the traumatized tooth, followed by prosthetic replacement with an implant or bridge, is indicated when the prognosis is very poor.

Four different types of conservative and endodontic treatment are recommended for the management of horizontal root fracture [15,16], as shown in Table 1.

**Table 1:** Types of conservative endodontic treatment. References [15,16]

Type	Endodontic Procedure
1	Endodontic treatment was limited to the coronal fragment, including cleaning and gutta-percha obturation.
2	Endodontic treatment, including cleaning and gutta-percha obturation in both the coronal and apical fragments.
3	Endodontic treatment in the coronal fragment and surgical removal of the apical fragment.
4	Chemomechanical preparation of coronal and apical fragments, filling the canal with calcium hydroxide dressing, followed by endodontic obturation.

In our case, we have decided to go for chemo-mechanical preparation of coronal and apical fragments, filling the canal with calcium hydroxide dressing, and endodontic obturation. Calcium hydroxide has antimicrobial and remineralizing actions [18]. A glass fiber post was used for anchoring both the fragments together, as it has high flexural, fatigue, and tensile strength. Moreover, it has a modulus of elasticity similar to that of dentin [13]. This similarity allows them to distribute stress more evenly under external forces, reducing the risk of root fracture. These features of resin-bonded fiber post make it clinically significant for its selection as a material of choice for radicular retainer (post and core). Additionally, their ability to bond effectively with tooth structure minimizes microleakage, and their aesthetic appearance makes them suitable for use in visually sensitive areas [13]. Following root canal therapy, fiber post placement, and tooth preparation, the tooth was restored and splinted with an all-ceramic bridge involving the adjacent teeth [19]. The tooth with CHRF is made out of occlusion in all mandibular masticatory functions to promote healing and repair.

Horizontal fracture repair would occur in this case presented, as hard tissue-forming cells would originate from the intact PDL, as evidenced in the existing literature [14]. Healing of horizontal root fracture undergoes four types of histological reaction [10,11] as shown in Table 2.

**Table 2:** Histologic responses. References [10,11]

Type	Histological Responses
I	Interposition of calcified tissue (callus formation) occurs when the coronal fragments are not displaced.
II	Interposition of connective tissue, which is characterized by the peripheral rounding of the fracture's ends.
III	Interposition of bone and connective tissue, radiologically characterized by the clear separation of the two fragments.
IV	Interposition of granulation tissue, caused by an infected or necrotic pulp.

Our case comes under the type I category, where the coronal fragment was not dislocated [15].

Prognosis is influenced by fracture location, time, pulp response, and patient factors. The more apical, the better the prognosis. Earlier management improves healing outcomes. Teeth with maintained vitality have better outcomes. Oral hygiene, systemic health, and compliance of the patient affect healing. A study by Budreikaitė et al. reported that there is a significant association between apical periodontitis and type 2 diabetes patients undergoing RCT [20]. Therefore, diabetic patients undergoing management for CHRF should be kept under long-term follow-up with regular recall visits and appropriate, timely interventions as needed. This patient is a controlled diabetic and is currently under medication, so proper healing of the CHRF is anticipated.

Studies have shown that up to 80% of mid-root fractures heal successfully without intervention. Cervical fractures have a lower success rate due to higher mobility and periodontal challenges. This paper presents a case report with CHRF and its management with an interdisciplinary approach, with a better prognosis even after one, three, and six months of recall visits. However, the limitations of this case report include a short-term follow-up period and the absence of CBCT imaging as a diagnostic tool. Therefore, further interventional study with long-term follow-up and inclusion of CBCT imaging technology should be included, which is required to describe the expected future outcome.

## Conclusions

CHRFs are complex injuries that demand careful diagnosis and an individualized treatment plan. While conservative, endodontic, and prosthetic management may preserve the tooth in some cases, surgical intervention might be required for long-term success. Diagnostic imaging, patient compliance, and close follow-up are key elements of successful management. Future advancements in regenerative endodontics and biomaterials may further enhance prognosis in cases of CHRF. However, future interventional studies with extended follow-up periods and the incorporation of CBCT imaging technology are recommended to better assess the long-term prognosis.
